# Photodynamic impact of curcumin enhanced silver functionalized graphene nanocomposites on *Candida* virulence

**DOI:** 10.1186/s11671-024-04017-5

**Published:** 2024-04-29

**Authors:** Dhivyabharathi Balakrishnan, Cheng-I Lee

**Affiliations:** 1 Department of Biomedical Sciences, National Chung Cheng University, Min-Hsiung, Chiayi, 62102 Taiwan, ROC; 2 Center for Nano Bio-Detections, National Chung Cheng University, Min-Hsiung, Chiayi, 62102 Taiwan, ROC; 3 Center for Innovative Research On Aging Society (CIRAS), National Chung Cheng University, Min-Hsiung, Chiayi, 62102 Taiwan, ROC; 4 Advanced Institute of Manufacturing With High-Tech Innovations, National Chung Cheng University, Chiayi, 62102 Taiwan, ROC

**Keywords:** Clinical *Candida* pathogens, Virulence, Pathogenicity, Carbon nanocomposites, Photodynamic treatment

## Abstract

*Candida* species are escalating resistance to conventional antifungal treatments, intensifying their virulence, and obstructing the effectiveness of antifungal medications. Addressing this challenge is essential for effectively managing *Candida* infections. The overarching objective is to advance the development of more efficient and precise therapies tailored to counter *Candida* infections. This study focuses on developing antifungal combined drugs using curcumin-enhanced silver-functionalized graphene nanocomposites (Cur-AgrGO) to effectively target key virulence factors of *C. albicans*, *C. tropicalis*, and *C. glabrata* (*Candida* spp.). The green reduction of graphene oxide (GO) using bioentities and active molecules makes this approach cost-effective and environmentally friendly. The nanocomposites were characterized using various techniques. Combining Cur-AgrGO with photodynamic therapy (PDT) demonstrated effective antifungal and antibiofilm activity with delayed growth and metabolism. The nanocomposites effectively suppressed hyphal transition and reduced key virulence factors, including proteinases, phospholipases, ergosterol levels, and cell membrane integrity. The findings suggest that Cur-AgrGO + PDT has potential as a treatment option for *Candida* infections. This innovative approach holds promise for treating *Candida* infections.

**Graphical abstract:**

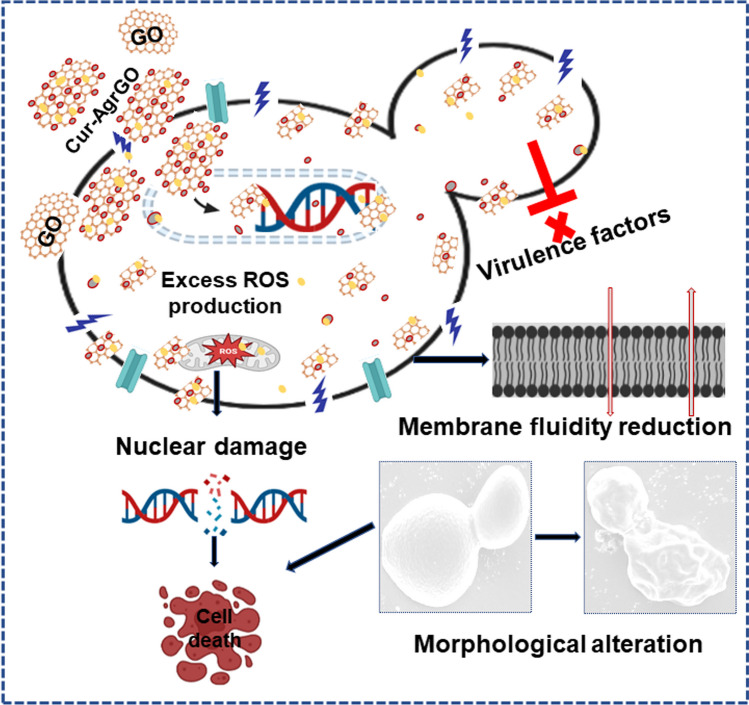

## Introduction

Fungal infections have become more common over the past few years; *Candida* species (spp.) is currently listed as the 4th leading root of nosocomial sepsis worldwide [1]. Over 1.7 billion infections are caused by 300 species of almost 1.5 million fungal species that exist worldwide. *Candida albicans* is the second-most common pathogenic agent among them [2]. An asexual and haploid *C. glabrata* and yeast-like fungi called *C. albicans* and *C. tropicalis* are present in the typical flora of the skin, vagina, mouth, and gastrointestinal tract of humans and cause infection in humans. They are frequently responsible for candidiasis [3]. One crucial aspect of *C. albicans* pathogenicity is its polymorphism, which enables revocable morphogenesis from the blastospores to a hyphal form [4]. *Candida* infections are resistant to many antifungal drugs, such as azoles and polyenes, such as ketoconazole and amphotericin B [5]. Pathogens in the food sector can also cause significant hygiene issues and even life-threatening illnesses. Specifically, it can result in infections linked to medical devices [6]. At this stage, antibacterial substances and agents are becoming increasingly crucial in the fight against microbes. However, due to widespread antibiotic use and the emergence of antibiotic-resistant microbial strains, conventional antibiotic therapies are no longer effective, leading to reduced treatment efficacy and sharply rising healthcare costs [7]. Numerous studies have confirmed the suppression of *Candida* spp. by evaluating the bioactive compounds alone and in combination with conventional antifungal drugs, specifically against *Candida* spp. impact on biofilm formation, which is vital for the virulence nature. However, it has been stated that this form of fungus is more challenging to eradicate than gram-positive and gram-negative bacteria [8]. Numerous studies state that to inactivate the fungus, more drugs with longer light exposure are necessary due to the existence of the cell membrane [9]. Delavy et al. [10] explored the resistance to colonization by *C. albicans*, a fungal pathogen known for triggering severe infections in individuals with weakened immune systems. By employing advanced OMIC-based techniques alongside experimental methodologies, the researchers sought innovative strategies to curb the proliferation of *C. albicans* within the microbiota and mitigate its detrimental effects. Therefore, highlighting the complexity of microbial interactions, it is becoming increasingly important to produce new generations of antimicrobial drugs to combat pathogens.

Nanomaterials have drawn much interest recently due to their potential applications in biomedical uses, including theranostics and imaging [11]. One of the most intriguing materials is two-dimensional (2D) layered nanomaterials because of their unique characteristics and large specific surface areas. Carbon atoms are organized in a single layer that resembles a honeycomb that makes up graphene, which has a remarkable array of physiochemical characteristics, including a high surface area, ideal thermal conductivity, notable optical transparency, and solid mechanical strength [12]. Owing to its greater properties, graphene opens up new interesting possibilities for the creation of novel therapeutic delivery systems, imaging techniques, and biosensor-based diagnostic instruments in nanomedicine [13]. A noteworthy example is the biological applications of graphene and its derivative graphene oxide (GO), particularly in microbial killing, DNA destruction, bioimaging, drug administration, photodynamic therapy, and photothermal therapy [14]. Silver and silver-based compounds have demonstrated potent antimicrobial activity against a diverse array of microorganisms, making them widely utilized in various bactericidal and fungicidal applications. In particular, silver nanoparticles (AgNPs) have emerged as effective and non-tolerant anti-infectants, playing a significant role in reducing numerous bacterial infections. Their exceptional antimicrobial properties and elevated microbial toxicity have garnered increasing attention in both biomedical and industrial fields among various nanomaterials. According to Halbandge et al., biosynthesized AgNPs have an impact on Ras-mediated signal transduction pathways in *C. albicans* by suppressing the activation of key genes responsible for yeast to hyphal transition (TUP1 and RFG1), the gene responsible for elongation of the cells (ECE1), and the gene inducing hyphae (TEC) [15]. The study conducted by Paloma Serrano-Díaz and colleagues investigated the OMIC-level antimicrobial properties of silver nanoparticles synthesized using geranium leaf extract. The findings on the OMIC profile suggest significant up- and down-regulation of genes essential for developing biofilm, cell adhesion, pathogenicity, virulence, and tissue penetration in *C. albicans* upon exposure to these silver nanoparticles, indicating potential antimicrobial properties [16].

Photodynamic therapy (PDT) is currently a major type of phototherapy widely used, and it includes the use of three major components: visible light, a photosensitive molecule, and an oxygen molecule [17]. Visible light is used to activate photosensitive molecules and tends to treat several diseases, including infections caused by fungi through reactive oxygen species (ROS) and singlet oxygen generation under light irradiation [18]. PDT has several benefits that can have longer-term negative effects than surgery and can be performed in a repeated manner on the area of target [19].

The natural bioactive constituents obtained from plant, animal, and microbial origins have been explored as potential photosensitizers (PSs) [20]. At present, several natural products, such as curcumin (Cur) and curcumin analogue, demethoxycurcumin, riboflavin, apigenin, resveratrol, hypericin, hypocrellin A, 8-methoxypsoralen, and psoralens, have been used in antimicrobial photodynamic therapy applications [21–23]. Table 1 summarizes the notable pharmacological effects of curcumin against various *candida spp*. The broad blue light absorption range (300–500 nm) of Cur makes it more significant as a potential natural PS to kill various fungal pathogens. Following an earlier report, it is clear that CUR-induced PDT action potentially results in significant cellular harm, cellular oxidative defense enzyme inactivation, and biological macromolecule oxidation [24].

**Table 1:** Literature studies investigating the effects of curcumin on *Candida* virulence

Pharmacologic effect	Research findings	References
Antifungal	Cur showed a potent antifungal inhibitory effect on nystatin‑resistant *C. albicans* strains	[25]
Antioxidant	Cur functions as a mimic of superoxide dismutase, potentially scavenging superoxides during stress-induced synthesis of ROS	[26]
Antibacterial	Facilitates intracellular ROS, which induces damage to the integrity and permeability of bacterial cell membranes	[27]
Antifungal	Cur has demonstrated a notable impact on genes that regulate cell wall integrity	[28]
Antifungal	An effective approach to overcome fungal infections, especially candidiasis	[29]
Antifungal	Excellent anti-adhesive and anti-biofilm against *C. parapsilosis*	[30]
Antifungal	Cur has dual effects on *Candida* cells, with early apoptosis leading to cell death and Cur also inhibits hyphae development by regulating TUP1 levels	[31]

Herein, we report a green synthesis of curcumin-enhanced silver-reduced graphene oxide (Cur-AgrGO) nanocomposites as a potential drug for antimicrobial PDT. In addition, antifungal properties and fungal pathogenic virulence factors were analyzed for three different *Candida* spp. A blue LED array was used as the photoactivation light source, and Cur in the Cur-AgrGO nanocomposite served as the photosensitizer. Curcumin-reduced graphene silver nanocomposites align with the core tenets of green biomaterials [32], owing to their biocompatibility, renewable source, eco-friendly synthesis, and enhanced properties. The application of Cur-AgrGO nanocomposites exemplifies the principles of green biomaterials by harnessing natural compounds, minimizing environmental impact, and offering sustainable solutions for biomedical applications.

## Materials and methods

### Clinical strains and culture conditions

Medical isolates of *Candida* spp. (*C. albicans*, *C. tropicalis*, and *C. glabrata*) were obtained from Dalin Tzu Chi General Hospital in Chia-Yi, Taiwan. To maintain the cultures, defrosted cells from a glycerol stock (30%) preserved at − 80 °C were streaked onto yeast extract peptone dextrose (YEPD) agar plates. These plates were then incubated at 37 °C for 24 h and subsequently stored at 4 °C for future utilization. *Candida* spp. at 1 × 10^6^ CFU/mL was used throughout the studies. YEPD broth was used as the routine medium for in vitro tests and cell cultivation. For in vitro tests related to biofilm formation and hyphal transitions, 10% fetal bovine serum (FBS) was added to the YEPD broth. The experiments were performed in triplicate for each test.

### Experiments of PDT

**Fig. 1:**
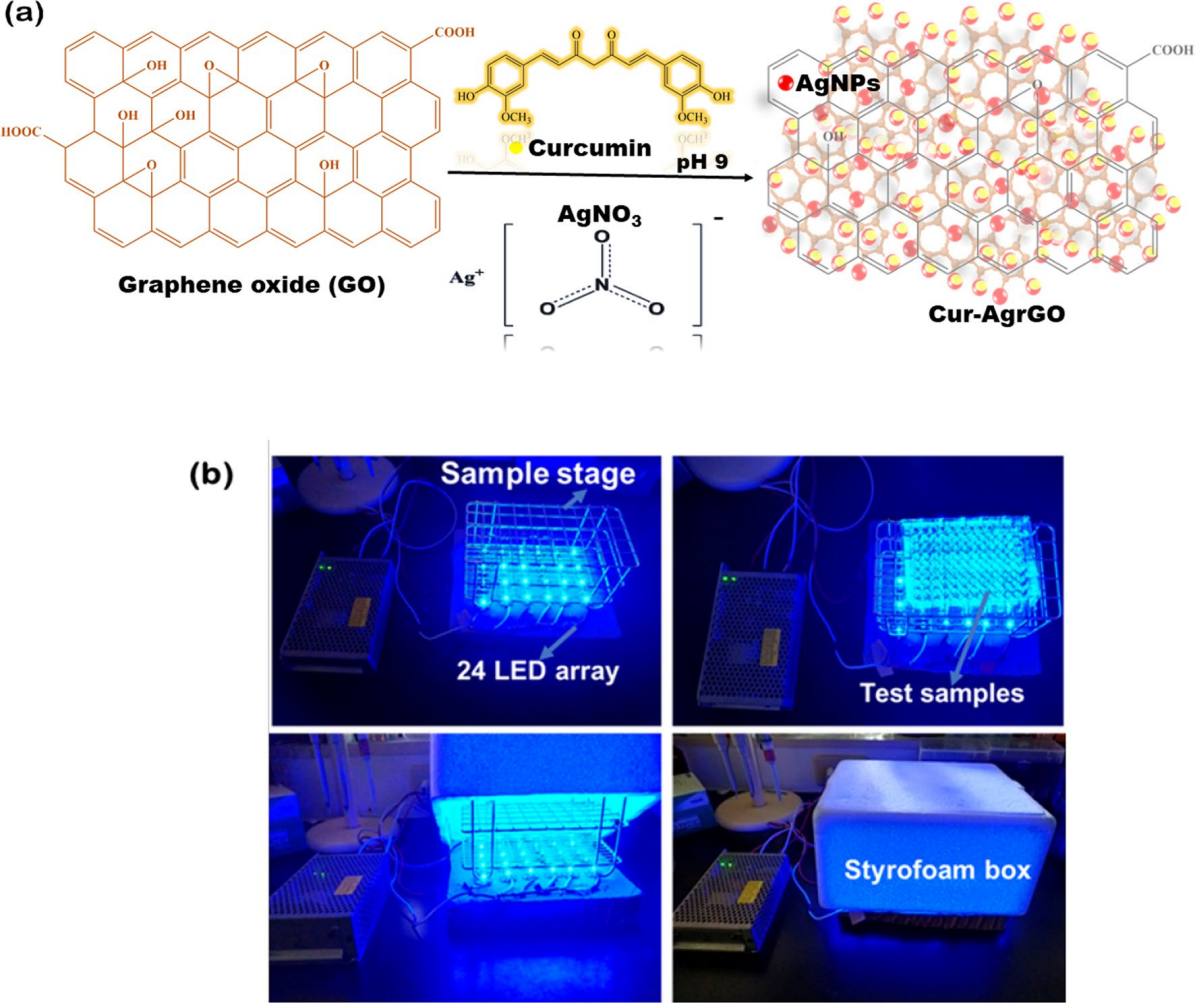
**a** A schematic illustration of the preparation of Cur-AgrGO from GO. **b** PDT device

The PDT experiments involved treating *Candida* spp. with Cur and Cur-AgrGO and exposing them to a custom device containing 24 blue LEDs emitting at 430 nm [33]. Figure 1b shows the PDT setup used in this research, (a) a device containing 24 blue LED arrays emitting at 430 nm, and (b) a chamber made from a Styrofoam box. PDT is performed by placing the LED array at the base and inversely covering the test sample stage with the styrofoam box chamber. After 30 min of illumination, a total of 9 J/cm^2^ of photoenergy was recorded.

### Synthesis of GO and Cur-AgrGO nanocomposites

Referring to our earlier published study [11], the graphite powder was first oxidized in a concentrated H_2_SO_4_ and KMnO_4_ mixture to yield graphene oxide (GO) using the modified Hummers process. To synthesize Cur-AgrGO nanosheets, 60 mg of GO was dissolved in 60 mL of deionized water (dH_2_O), and 5 mM AgNO_3_ was dissolved in 30 mL of dH_2_O. These solutions were combined and stirred continuously for 20 min. To the above mixture, 1 mM Cur was then incorporated. The resulting mixture was maintained at 90 °C for one hour with constant stirring. Upon completion of the reaction, the nanocomposites were centrifuged for 10 min at 10,000 rpm. Finally, the nanocomposites were lyophilized and characterized for further use.

### Physio-chemical characterization of nanocomposites

The nanocomposite functional groups were analyzed using an FTIR spectrometer (Bruker VERTEX 70 V, Norwalk, CT, USA). Potassium bromide (KBr) was mixed with 2 mg of powdered samples of GO, Cur, rGO, and Cur-AgrGO, and the resulting pellets were analyzed between 400 and 4000 cm^−1^. UV–visible spectroscopy (SCINCO S-3100, Seoul, Korea) in the range of 200 to 800 nm was performed. Transmission electron microscopy (TEM, model JEOL JEM 1210, Tokyo, Japan) and a field emission-scanning electron microscope with an attached EDX (FE-SEM/EDX, Hitachi S4800-I, Tokyo, Japan) analysis were performed to acquire the nanoparticle morphology. Raman spectra were recorded using laser confocal micro-Raman spectroscopy (Horiba Scientific XpoloRA, Kyoto, Japan). On a Zetasizer analyzer, zeta potential analysis was carried out (Malvern Instruments Ltd., Worcestershire, UK). Fluorescence images were captured using a fluorescence microscope (Nikon, Eclipse 80i; Tokyo, Japan).

### Yeast to hyphal transition inhibition

The morphogenesis assay was performed according to the protocol of Silva-Das et al., with slight modifications [34]. To assay the transition from yeast to hyphae, cells were cultured overnight at 30 °C, and the pH was maintained at 6. Cells were collected from a 12–24 h grown culture and supplemented with 10% FBS, and incubation was carried out for 4 to 6 h at 37 °C. To evaluate the effect of the nanocomposites on morphogenesis, test materials (GO, rGO, Cur, Cur + PDT, Cur-AgrGO, Cur-AgrGO + PDT) and fluconazole (Flu) positive control (20 µL) were also added to the medium. After 4 h of treatment, the cells were photographed by an inverted microscope (Zeiss Axiovert 200 M, Thornwood, NY). The cell volume and diameter were measured by ImageJ software (National Institutes of Health, Bethesda, Maryland, USA).

### Time-course growth inhibition assay

*Candida* cells at 1 × 10^6^ cells/mL were treated with 20 µL of the test materials and fluconazole, and the tubes were kept at 30 °C with 150 rpm agitation. A fixed volume of aliquots was taken from each test tube at fixed time intervals of 0, 2, 4, 6, 12, 24, and 48 h and washed three times with dH_2_O. The washed pellet was suspended in 0.1 mL of distilled water and further serially diluted in distilled water, and 10 µl was taken from this suspension and spread on YEPD agar plates by a spreader [35]. The plates were incubated at 30 °C for 48 h. The results were recorded as the number of colonies (CFU) formed after incubation for 48 h.

### Antifungal activity and morphological changes *in Candida* spp.

The minimum inhibitory concentration (MIC) of the anticandidal nanocomposites was tested against three *Candida* spp. by the microbroth dilution method and quantified [36]. The anticandidal properties of the nanocomposites at MICs without light and with PDT exposure were checked by calculating the zone of inhibition (mm) on Sabouraud dextrose agar (SDA) plates by the Kirby–Bauer Clinical and Laboratory Standards Institute (CLSI) antimicrobial susceptibility disc diffusion method [37]. *Candida* cells were supplemented with the IC_90_ of Cur-AgrGO and Cur-AgrGO + PDT for 12 h. Cells were collected by centrifugation at 1500 rpm for 10 min and fixed in 2% glutaraldehyde in 0.1 phosphate buffer. Glutaraldehyde-fixed samples were observed through FE-SEM (HITACHI SU-5000, Tokyo, Japan).

### Biofilm and adhesion assay

Overnight-grown *Candida* cells at 1 × 10^6^ cells/mL were collected by spinning and suspended in PBS. Test materials and fluconazole (20 µL) were added to 96-well plates containing pre-inoculated FBS overnight. A 200-μl cell suspension was added to the FBS-treated plates and incubated at 37 °C for 48 h in a gentle shaking condition. After rinsing the cell wells with PBS, biofilms were stained with crystal violet (CV) for 30 min. CV was dissolved using ethanol, and the absorbance of the eluate at 560 nm was measured to quantify biofilm formation.

### Determination of singlet oxygen (^1^O_2_)

By observing the quenching of DPBF fluorescence, the singlet oxygen generation capacity was assessed [38]. For a total volume of 800 μl, 50 μl of 10 μM DPBF was first combined with 750 μl of 2 mg/mL Cur-AgrGO. The measurement excitation wavelength is set at 403 nm, and the excitation and emission grating slits are 10 nm. The sample is then illuminated with LED blue light for 0, 1, 5, and 10 min, after which measurements are taken every 10 min. The test was halted after 40 min, during which the fluorescence value did not significantly change. After 40 min of light illumination, 50 μl of 10 μM DPBF was combined with water for the control group, and it was compared with no illumination.

#### Photobleaching test of curcumin

To determine the photobleaching effect of Cur in Cur-AgrGO, 1 mg/mL of Cur-AgrGO was diluted in dH_2_O. The sample was illuminated with LED blue light at different time intervals of 1, 5, 10, 20, 40, and 80 min. O.D. values were measured using a spectrophotometer for 80 min [39].

#### Effect of nanomaterials on *Candida* spp. plasma membrane by DPH fluorescence

To assess changes in the membrane surface, we examined the fluorescence intensity of the *Candida* cytoplasmic membrane labeled with 1,6-diphenyl-1,3,5-hexatriene (DPH) [40]. *Candida* cells were exposed to 20 μl of various test materials and fluconazole. Following a 2-h incubation, the cells were fixed in formaldehyde (0.37%), washed, and stored at -80 °C. Thawed cells were treated with DPH (0.6 mM) and incubated for 45 min at 28 °C. After washing and sonication, the DPH-labeled lipid matrix in the supernatant was analyzed in a spectrofluorometer (excitation at 350 nm, emission at 425 nm).

#### Effect of Cur-AgrGO determined by propidium iodide staining

*Candida* cells were treated with 20 μl of GO, Cur-AgrGO, and Cur-AgrGO + PDT. Following treatment, the cells were washed with PBS, fixed in 70% ethanol for 30 min at 4 °C, and washed in PBS. Then, the cells were suspended in 50 μl of RNase and incubated at 37 °C for 60 min. The cells were washed in water with initial rinses using PBS and ethanol. Finally, the cells were stained with 200 μl of propidium iodide (PI) for 1 h and examined under a fluorescence microscope after the designated time [41].

#### Quantification of ergosterol

*Candida* cells treated with 20 μl of test materials were centrifuged to obtain cell pellets and weighed. Alcoholic KOH was added to the pellet and maintained at 50 °C for 30 min. Distilled water and heptane in a 1:3 ratio were added to each tube for sterol extraction. The tube was vortexed to obtain a separated heptane layer. The heptane layer was collected in a new tube and diluted five-fold with absolute ethanol. The UV spectrum of this layer was obtained at 240–300 nm with respect to the ethanol blank. A characteristic curve with four peaks confirms the existence of ergosterol. The Arthington-Skaggs procedure was used to calculate the percentage of ergosterol in the sample [42].

#### Estimation of hydrolytic enzyme secretion

The secretion of hydrolytic enzymes was studied by the method of Eladly et al. with slight modifications [43]. *Candida* spp. was treated with 20 μl of test materials and fluconazole. Cells were maintained at 30 °C for 18 h with 150 rpm agitation. Following incubation, the cells were spun at 5000 rpm for 5 min, and the pellet was dissolved in normal saline. Tubes were agitated for 2 min on a vortex mixer, and 2 µl from each tube was spotted on proteinase agar plates and phospholipase agar plates. The plates containing inoculates for the detection of proteinase secretion and phospholipase secretion were incubated for 24 h and 96 h, respectively, at 37 °C. Proteinase secretion was determined by the degradation of bovine serum albumin (BSA), while the presence of phospholipase secretion was determined by the degradation of egg yolk. Secretions of hydrolytic enzymes by *Candida* spp. were determined by measuring the colony diameter by the zone of degradation diameter, which includes the diameter of the colony.

#### Statistical analysis

GraphPad Prism is used for statistical analysis and graphing in this research. A student's t-test was conducted for statistical comparisons between two sets of data. A p-value of < 0.05 was considered to be statistically significant.

## Results and discussion

### Synthesis of GO and Cur-AgrGO nanocomposites

A green route for graphene oxide synthesis was reported in our previous study [11]. We developed Cur-AgrGO nanosheets by initially incorporating silver (Ag) ions onto graphene sheets and subsequently employing curcumin for their reduction. In this current investigation, a novel approach was employed where Ag ions were concurrently reduced and loaded onto GO nanosheet surfaces. These Ag ions were then stabilized through polyphenolic functionalization, leading to the creation of Cur-AgrGO nanocomposites. Figure 1a illustrates the process of environmentally friendly reduction of GO through the utilization of curcumin. Silver ions, upon reduction with the curcumin reducing agent, result in the formation of spherical silver nanoparticles and are deposited onto the surface of the graphene oxide sheets. This approach mitigates the adverse consequences associated with the use of harmful reducing agents. The interaction between oxidized curcumin and GO involves the hydroxyl and carbonyl groups of curcumin forming strong hydrogen bonds with the remaining oxygen functionalities on the GO surface. This unique bonding mechanism allows curcumin to serve as a stabilizing agent for the resulting Cur-AgrGO products. Additionally, the oxygen functional groups of curcumin on the surface of Cur-AgrGO induce electrostatic repulsion between the particles, effectively enhancing stability and preventing undesirable aggregation. As a result, this novel interaction offers promising prospects for various applications. Furthermore, successful synthesis was confirmed by physio-chemical characterization.

### Physio-chemical characterization of nanocomposites

**Fig. 2:**
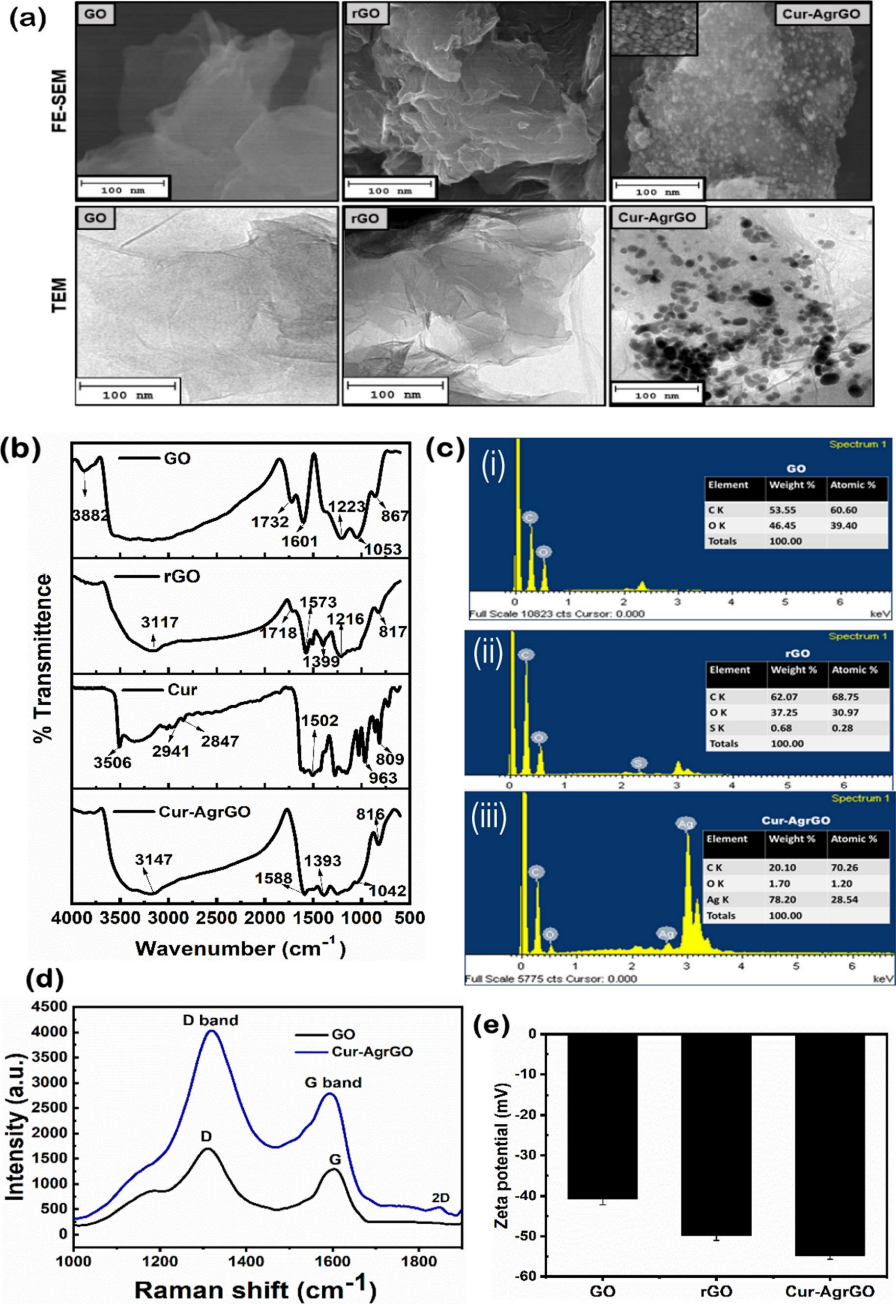
**a** FE-SEM and TEM analysis of GO, rGO, and Cur-AgrGO. **b** FTIR spectra of GO, rGO, Cur, and Cur-AgrGO. **c** EDX analysis of GO, rGO, and Cur-AgrGO. **d** Raman spectra of GO and Cur-AgrGO. **e** Zeta potential values of GO, rGO, and Cur-AgrGO

The FE-SEM and TEM micrographs presented in Fig. 2a provide valuable insights into the morphological characteristics of GO, rGO, and Cur-AgrGO nanocomposites. These images depict the typical sheet-like structure of GO, with rGO exhibiting a thinly crushed and slightly translucent morphology. Incorporation of curcumin as a reducing agent in the synthesis of Cur-AgrGO nanocomposites exhibits almost homogeneous, spherical-shaped well-dispersed AgNPs with sizes ranging from 15 to 30 nm anchored to the surface of GO. This observation underscores the effectiveness of curcumin in facilitating the reduction of Ag ions and the subsequent formation of AgNPs on the surface of graphene oxide. The controlled size of AgNPs in Cur-AgrGO highlights the successful synthesis of a nanocomposite with potential applications.

In Fig. 2a, owing to the existence of hydroxyl and carbonyl groups, the exfoliation of graphite into GO was seen to be a flaky and extremely transparent layer in the TEM micrographs. The rGO surface produced a structural rupture with raised crumples and stacking wrinkles upon reduction. The rough surface thus increases its hydrophobic nature. The Cur-AgrGO TEM data demonstrate that a significant amount of small silver nanoparticles with good dispersion were integrated onto the GO surface. The results showed that curcumin not only converts silver salt to AgNPs but also strongly caps and stabilizes the newly generated AgNPs on the GO surface, stopping them from aggregating without the need for further support.

Figure 2b shows the FTIR spectra of GO, rGO, Cur, and Cur-AgrGO. The vibrations for free Cur were observed at 3506, 2941, 2847, 1502, 963, and 809 cm^−1^. Regarding GO, vibrational stretches related to epoxy, alkene C=C, and hydroxyl O–H were observed at 1601 cm^−1^, 1053 cm^−1^, and 3882 cm^−1^, respectively. The carboxylic groups (1732 cm^−1^) decreased after the reduction of GO by Cur, with newly appearing peaks at 1573 and 1399 cm^−1^ indicating C=C bond repair. The C–O–C stretch of Cur-AgrGO represented a low-intensity band at 3147 and 1042 cm^−1^. Comparing GO and Cur-AgrGO, significant deoxygenation in carboxyl and epoxide groups was observed. Differences in vibrational band intensities supported contact and capping of Cur onto the GO nanosheets.

An EDX analysis provided additional proof that graphene oxide had been reduced and that silver nanoparticles had been successfully deposited onto the surface of the reduced graphene oxide nanosheets. The compositions of the elements present in GO, rGO, and Cur-AgrGO are shown in Fig. 2c(i)–(iii). Signals for carbon and oxygen on the surface of GO were detected in Fig. 2c(i). The reduction in the oxygen content in rGO showed that oxygenated functional moieties had been reduced. Figure 2c(ii) shows that the hydrophilic graphene also underwent a hydrophobic transformation. Numerous intense Ag peaks in the elemental composition of Cur-AgrGO showed that the reduced graphene oxide surface had integrated and absorbed a larger concentration of monodispersed silver nanoparticles, as shown in Fig. 2c(iii).

Raman spectroscopy is an effective method for analyzing disordered sp^2^ carbon materials. The GO spectrum in Fig. 2d shows the presence of the D band (E_2g_ mode) at 1309 cm^−1^ and the G band (A_1g_ mode) at 1604 cm^−1^, representing phonons with specific symmetries related to the breathing and tangential stretching modes of carbon atoms. The high purity of the GO used is indicated by a D/G intensity ratio of 1.3. The incorporation of curcumin silver nanoparticles introduces structural flaws, evident in the 2D peak. AgNP incorporation on the GO surface alters the intensity of the D and G bands in the Cur-AgrGO spectra at 1320 cm^−1^ and 1598 cm^−1^, respectively. Following polyphenolic functionalization, the D/G intensity ratio of Cur-AgrGO increased from 1.3 to 1.5. Sheet stacking affects the 2D band in Raman spectroscopy of graphene materials.

The zeta potentials of the GO, rGO, and Cur-AgrGO colloids were measured. Figure 2e shows that the GO suspension has a zeta potential of − 40.6 mV, which denotes that the existence of hydrophilic carboxyl groups results in negatively charged surfaces. The reduction and deposition of negatively charged silver nanoparticles on the surface of GO caused the zeta potential of the rGO and Cur-AgrGO composites to become more negative at − 49.7 mV and − 54.7 mV, respectively, with the addition of curcumin and silver nanoparticles.

This detailed characterization of morphological features provides a foundation for understanding the structure–property relationships and elucidating the potential mechanisms underlying the functional properties of Cur-AgrGO nanocomposites.

### Yeast to hyphal transition inhibition

**Fig. 3:**
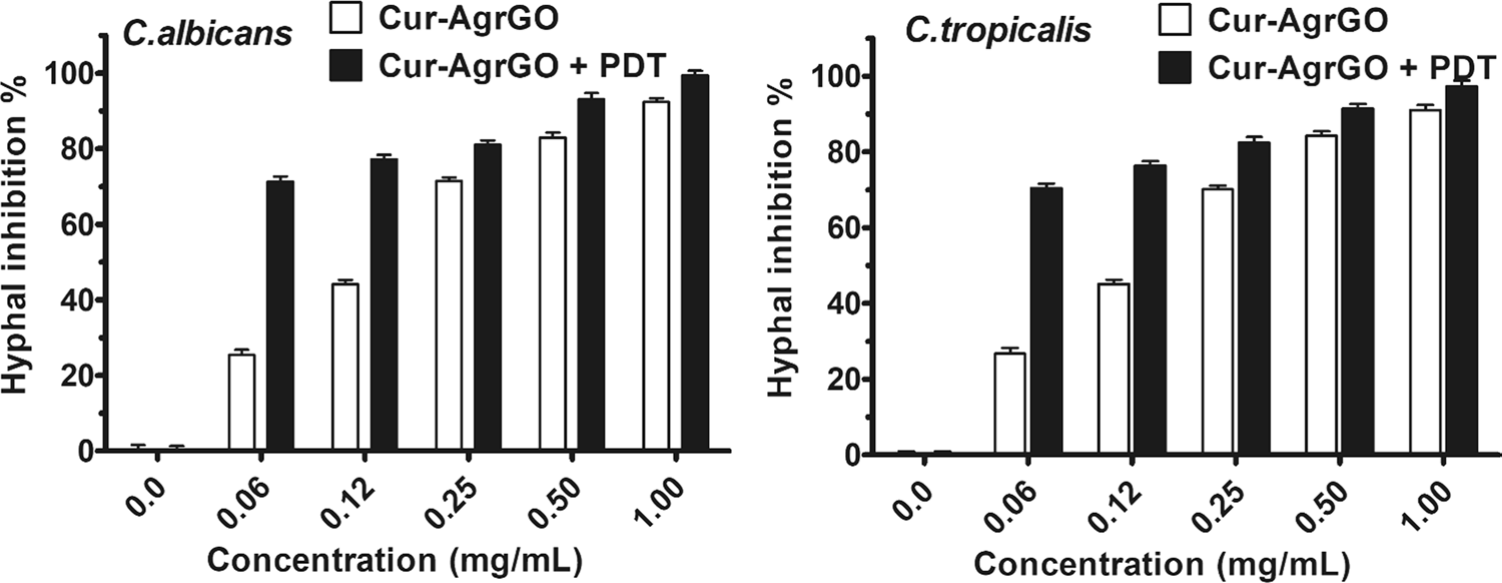
Inhibition of the yeast-to-hyphal transition in *C. albicans* and *C. tropicalis* treated with Cur-AgrGO and Cur-AgrGO + PDT

Germ tube development and mycelial hyphae formation are vital aspects of *Candida* spp., contributing to attachment, biofilm development, and colonization, which are key factors in their pathogenicity [44]. This study delved into the impact of biosynthetic Cur-AgrGO and Cur-AgrGO + PDT treatments on germ tube formation, revealing significant inhibition of morphological transitions (Fig. 3). The results showed a reduction in germ tube formation compared to untreated cells. In *C. albicans*, Cur-AgrGO at concentrations of 1, 0.5, 0.25, 0.12, and 0.06 mg/mL reduced germ tube formation by 88.3%, 80.2%, 71.4%, 44.1%, and 25.4%, respectively. PDT treatment at the same concentrations further increased the inhibition to 99.4%, 93.1%, 81.01%, 77.2%, and 71.3%. *C. glabrata* exhibited complete growth suppression without hyphal transition post-treatment. *C. tropicalis* displayed substantial reductions with Cur-AgrGO of 91.1%, 84.2%, 70.1%, 45.1%, and 26.1%. The combination of Cur-AgrGO and PDT showed even higher inhibition rates, with reductions of 97.3%, 91.4%, 82.4%, 76.4%, and 70.4%. These findings demonstrate the potent inhibitory effects on germ tube formation by Cur-AgrGO and Cur-AgrGO + PDT.

### Time-course growth inhibition assay

**Fig. 4:**
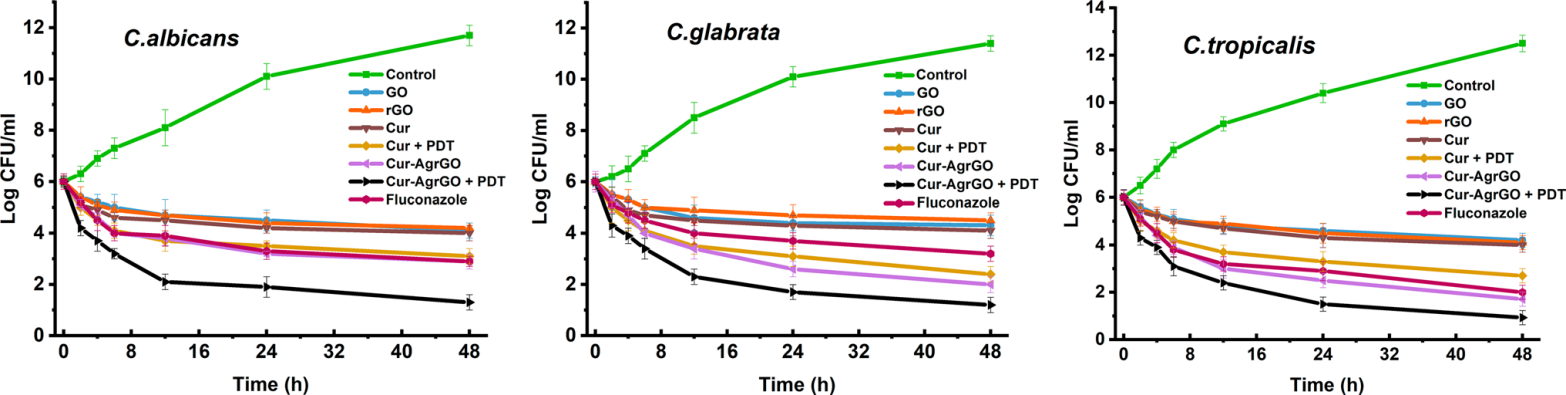
Time-dependent growth curve of *Candida* spp. treated with GO, rGO, Cur, Cur + PDT, Cur-AgrGO, Cur-AgrGO + PDT and fluconazole

Figure 4 offers compelling insights into the dynamic growth kinetics of *Candida* spp. and the inhibitory effects of Cur-AgrGO and PDT. Notably, the net growth rates dropped sharply after 2 h, and a considerable decline in the number of *Candida* cells was observed between 2 and 6 h of exposure. Remarkably, over 48 h of incubation at 37 °C, Cur-AgrGO and Cur-AgrGO + PDT interestingly showed better inhibition activity than fluconazole in terms of the growth of the yeast cells. The nanocomposite and PDT demonstrated the most effectiveness against *C. tropicalis* in the assay. The assay also revealed that Cur-AgrGO nanocomposites and Cur-AgrGO + PDT displayed the most robust inhibition kinetics, with no observed regrowth of *Candida* cells. The results of the time-dependent growth inhibition experiment showed that GO, rGO, Cur, Cur + PDT, Cur-AgrGO, Cur-AgrGO + PDT, and fluconazole have strong anticandidal properties based on changes in the CFU of viable *Candida* colonies.

### Antifungal activity and mechanism of action

**Fig. 5:**
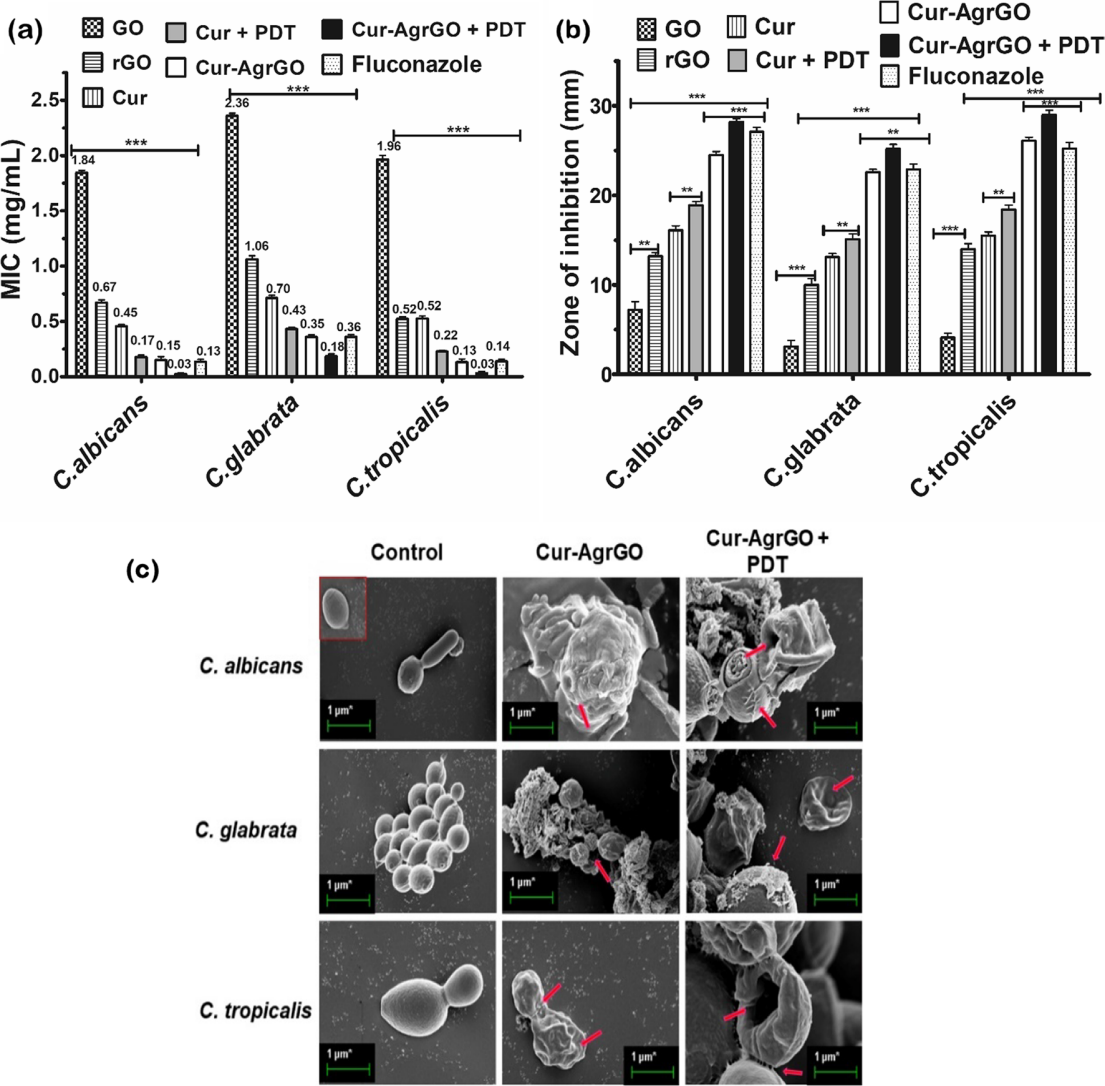
Antifungal activity **a** MIC of *Candida* spp.; **b** zone of inhibition of *Candida* spp. treated with GO, rGO, Cur, Cur + PDT, Cur-AgrGO, Cur-AgrGO + PDT and fluconazole; (the ** represents *p* < 0.001 and *** represents *p* < 0.0001). **c** FE-SEM structural changes of *Candida* spp. treated with Cur-AgrGO, and Cur-AgrGO + PDT (red arrows represent the nanocomposites wrapped on *Candida* spp. and structural damage)

The MICs of the test materials were calculated and are shown in Fig. 5a. The MIC range of *C. tropicalis* was similar to that of *C. albicans,* and the tested antifungal agents were more effective at their lower concentrations. According to some studies, *C. albicans* is more genetically and phenotypically related to *C. tropicalis* than to *C. glabrata.* In the case of *C. glabrata,* the MIC was found to be greater than that of the other two *Candida* spp.

The antifungal properties of the tested nanomaterials were evaluated by measuring the zone of inhibition on SDA plates (Fig. 5b). The maximum inhibition was observed for *C. tropicalis* treated with Cur-AgrGO + PDT (29 mm), followed by *C. albicans* treated with Cur-AgrGO + PDT (28.2 mm), and *C. tropicalis* treated with Cur-AgrGO (26.1 mm). The maximum inhibition for *C. glabrata* was recorded at 25.6 mm Cur-AgrGO + PDT. The inhibition zone increased with PDT; comparatively, the tested antifungal materials were less effective against *C. glabrata*. Despite having similar MIC values as *C. albicans* and *C. tropicalis* in SDA, *C. glabrata* is less sensitive to antifungals, most likely because of its invasive morphological form.

A stable colloidal system was attained by incorporating the Cur-AgrGO suspension into water, increasing its contact area with microorganisms [45]. GO, possessing a vast surface area and a variety of functional groups such as (–O–), (–OH), (–COOH), and (–C = O), was capable of interacting with the cell membrane through hydrogen bonding and electrostatic forces, aiding in microorganism entrapment. Subsequently, Ag nanoparticles (AgNPs) release Ag^+^ ions into the mixture, enabling these ions to establish electrostatic bonds with phospholipids on the cell membrane. The sharp edges of GO sheets presented a potential threat to the integrity of the cell membrane [46]. The interaction between Ag^+^ ions and phospholipid bilayers led to a modification in the permeability of the cell membrane, a critical aspect of metabolic and transport processes. As a result, intracellular compounds were able to escape when cell disruption allowed Ag^+^ ions to penetrate. The introduction of Ag^+^ ions into the inner structure of cells could also induce changes and denaturation in DNA. Ag^+^ ions tend to react with thiol groups in amino acids, potentially impeding protein production. Additionally, the presence of Ag^+^ ions gave rise to the generation of ROS, which caused damage to microbial cells and their living systems.

#### Morphological changes of *Candida* cells exposed to nanocomposite-PDT

FE-SEM analysis was conducted to examine the impact of Cur-AgrGO and its photodynamic effect on the structural integrity of *Candida* spp., as illustrated in Fig. 5c. The observed cells exhibited a destructive morphology when treated with the test compounds Cur-AgrGO and Cur-AgrGO + PDT. Direct contact with the graphene oxide nanosheets led to physical destruction and cell death. The additional deformities induced by PDT, such as cytoplasmic leakage, emphasize the synergistic effect of combining Cur-AgrGO with light activation. In contrast, untreated cells remained unchanged in morphology, underscoring the specificity of the treatment's impact on fungal cells. The FE-SEM analysis further elucidated the mechanism of action, revealing that Cur-AgrGO + PDT effectively targeted the cell membrane, penetrated the cells by rupturing the cell wall, and accumulated in the cytoplasm. This accumulation ultimately led to the disintegration of the cytoplasmic membrane, further compromising the structural integrity of the fungal cells (Fig. 5c, indicated by red arrows). These findings align with the antimicrobial effects observed in the spread plate disc diffusion assays for Cur-AgrGO against *Candida* spp. Vazquez-Muñoz et al. suggested that the use of graphene nanocomposites inhibits morphological transitions and induces early-stage cell death in *Candida* cells [47].

### Biofilm inhibition assay

**Fig. 6:**
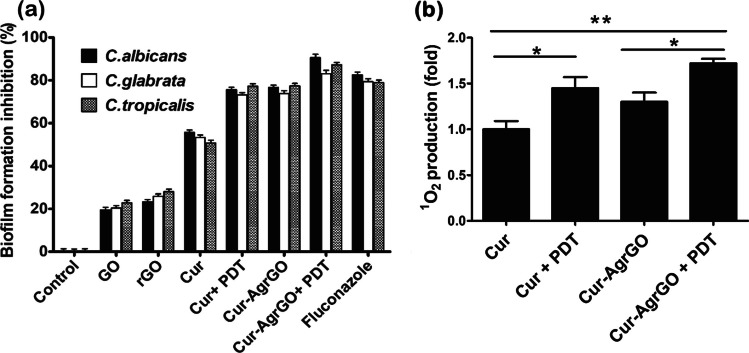
**a** Quantification of biofilm inhibition; **b** Determination of singlet oxygen generation (the * represents *p* < 0.05 and ** represents *p* < 0.001)

Biofilm formation is a crucial virulence factor in *Candida* spp., enabling colonization on various medical devices such as prostheses, catheters, and pacemakers [48]. Researchers have explored different anticandidal materials to hinder biofilm formation. Figure 6a illustrates biofilm quantification using CV staining after 48 h of treatment with anticandidal materials and PDT. The control group exhibited prominent biofilm matrix formation, while surfaces treated with GO, rGO, and Cur showed slight prevention of biofilm formation. Notably, Cur-AgrGO significantly reduced early and mature biofilm formation by 60–70%. In comparison, Cur + PDT and Cur-AgrGO + PDT treatments demonstrated extensive inhibition, with biofilm formation rates marked at 75% and 97%, respectively. Overall, the results indicate that Cur-AgrGO + PDT inhibits cell attachment, leading to dead cells with weaker biofilm formation ability on GO, rGO, and Cur surfaces. Additionally, photoactivation with blue LEDs for 15 min resulted in an over 95% reduction in *Candida* cell viability. This indicates the extensive inhibition of microcolony formation, filamentation, monolayer development, and cell proliferation after cell adherence. Similarly, AgNPs show a targeted action on the extracellular matrix (ECM) of *Candida* biofilms, comprising polysaccharides, proteins, and various macromolecules. This targeted effect leads to a weakening of the biofilm’s structural integrity, rendering it more vulnerable to antimicrobial agents [49].

### Singlet oxygen generation

According to Fig. 6b, when mixed with Cur-AgrGO nanocomposites and subjected to blue LED at a power density of 1.5 W cm^2^, DPBF absorbance rapidly fell by 75% within one minute, showing strong ^1^O_2_ production. The absorbance of DPBF remained essentially unaltered in the control tests, which did not use blue light illumination or DPBF exposure, indicating the lack of ^1^O_2_. These findings distinctly demonstrate that energy transfer from Cur-AgrGO nanocomposites during PDT exposure was responsible for initiating the production of ^1^O_2_, demonstrating the enormous potential of the Cur-AgrGO nanosystem as a powerful light-triggered antimicrobial PDT agent.

### Photobleaching test of curcumin

**Fig. 7:**
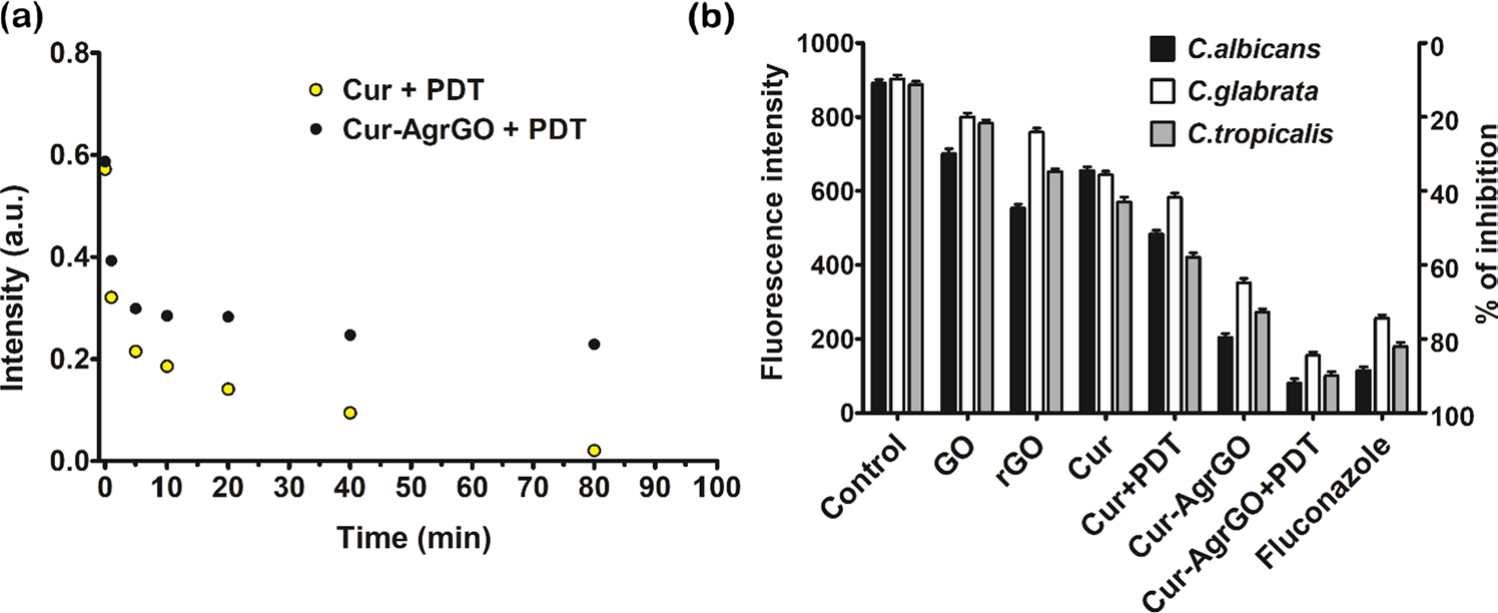
**a** Photobleaching analysis of curcumin in the Cur-AgrGO nanocomposite. **b** Fluorescence intensity of *Candida* spp. plasma membrane labeled with DPH

The AgrGO nanocomposite represents a promising strategy for shielding curcumin from photobleaching, a common challenge in PDT applications. Photobleaching typically occurs when photosensitizers, like curcumin, react with ROS, forming oxygenated adducts on their molecular structure [50]. In comparison to free curcumin, the intensity of photobleaching diminishes notably within a shorter timeframe during PDT exposure. However, in Cur-AgGO nanocomposites, the photostability of curcumin remains relatively preserved (Fig. 7a), indicating that AgrGO effectively shields curcumin from photobleaching. The unique structure of GO in the AgrGO nanocomposite offers multiple mechanisms to counteract photobleaching. Due to their interactions, the graphene sheets in this situation shield 50% of the photosensitizer surface area, protecting the curcumin molecules. Moreover, the large surface area of graphene allows for rapid interaction with ROS, serving as a hiding spot for curcumin molecules and preventing their degradation. This robust protective effect of AgrGO against photobleaching highlights its potential as a promising platform for enhancing the stability and efficacy of curcumin-based PDT therapies.

### Effect of nanomaterials on *Candida* spp. plasma membrane labeled with DPH

The results from DPH fluorescence intensity assays shed light on the membrane-disrupting effects of various treatments on *Candida* spp. DPH, a hydrophobic membrane probe [51], binds to phospholipids within the cytoplasmic membrane, enabling the assessment of membrane polarization. Figure 7b illustrates the decrease in DPH fluorescence intensity of treated cells, indicative of membrane destabilization. The relative percentages of DPH binding to *C. albicans* cells and their inhibition rates after a 30-min treatment of control, GO, rGO, Cur, Cur + PDT, Cur-AgrGO, Cur-AgrGO + PDT, and fluconazole were recorded as 11, 30, 41, 36, 59, 72, 92, and 81%, respectively. For *C. glabrata* cells, the membrane polarization and growth inhibition rates were measured at 11, 20, 37, 42, 63, 84, and 78%. Regarding *C. tropicalis* cells, the intensities were 10.1, 21, 33, 44, 48, 77, 90, and 89%, respectively. These results indicate that the tested materials, along with PDT, exhibit greater antifungal effects on all three *Candida* cell types by affecting the structure of the membrane lipid bilayer. Overall, these findings highlight the therapeutic potential of Cur-AgrGO nanocomposites and PDT as innovative strategies for combating *Candida* infections by disrupting membrane integrity and warrant further investigation into their clinical applications.

#### Effect of Cur-AgrGO on *Candida* spp. survival using PI

**Fig. 8:**
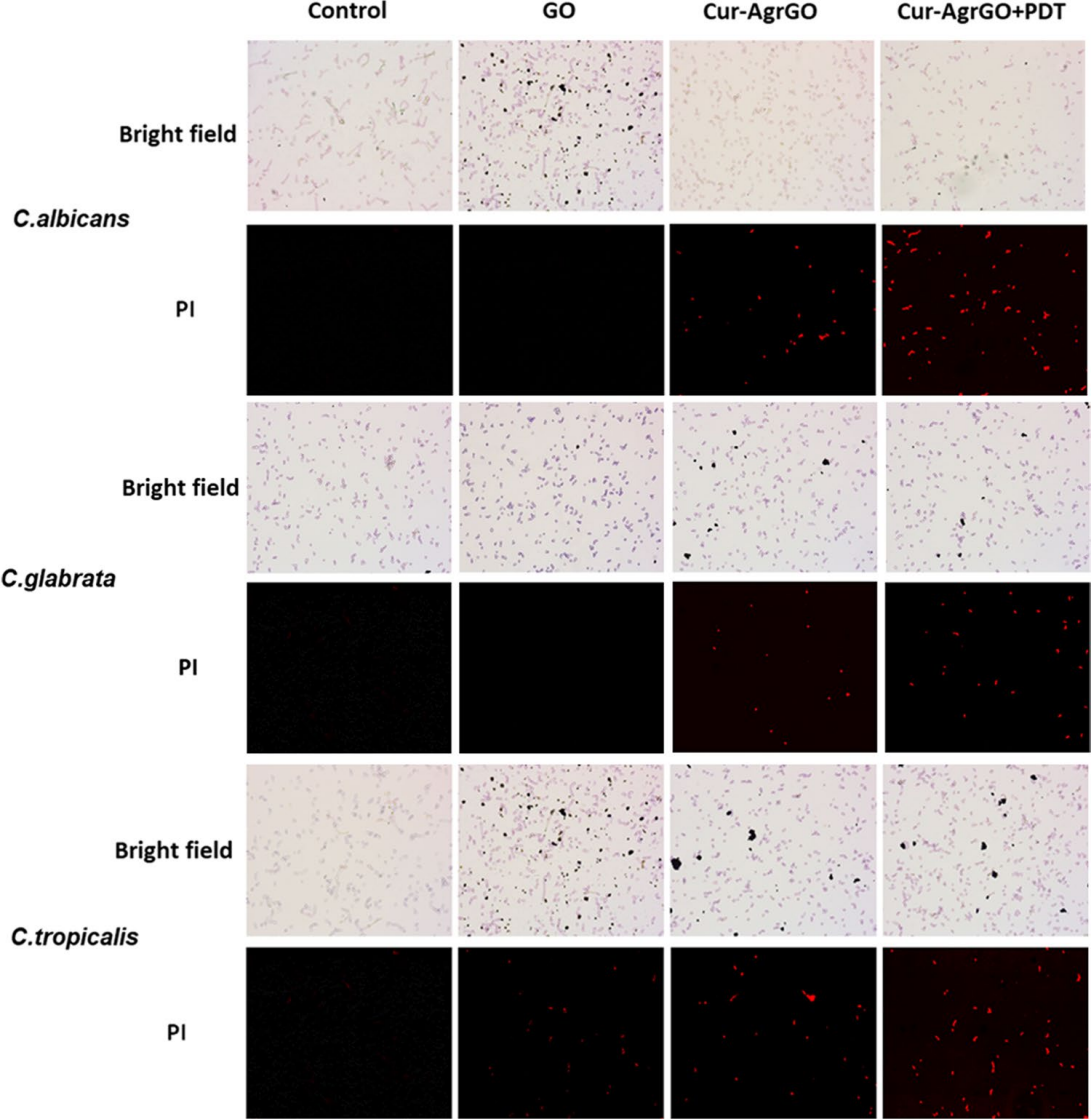
Effect of GO, Cur-AgrGO, and Cur-AgrGO + PDT on PI-stained *Candida* spp

Plant-derived substances are known for their ability to disrupt cell membrane integrity, leading to membrane-active action [52]. By utilizing the fluorescent dye PI, we investigated the effects of test materials on the integrity of cell membranes. Fluorescence microscopy was used to observe the uptake of PI by *Candida* cells in the presence of test materials at inhibitory concentrations. Here, Cur-AgrGO and Cur-AgrGO + PDT damaged all three *Candida* spp. cell membranes, as shown by PI staining, as shown in Fig. 8. When compared to untreated cells, Cur-AgrGO and Cur-AgrGO + PDT treated cells showed a higher degree of red fluorescence. This outcome demonstrated that the nanocomposite treatment harmed the fungal cell membrane. Importantly, our research elucidated the diffusion of PI within *C. tropicalis* cells through the damaged plasma membrane, providing insights into the mechanisms underlying the action of nanocomposite PDT on *Candida* cell membranes. These findings suggest avenues for further exploration in the development of novel antifungal therapies.

#### Quantification of sterols

Lipids play crucial roles in regulating membrane permeability and fluidity [53]. Among various types of fungal lipids, membrane ergosterol holds particular significance, as it is a key component of fungal cell membranes. Notably, many commonly used antifungal drugs target the ergosterol synthesis pathway (such as azoles, polyenes, and allylamines). This study aimed to investigate how Cur-AgrGO and Cur-AgrGO + PDT affect *Candida* spp. cell membranes by examining their impact on lipid composition.

**Fig. 9:**
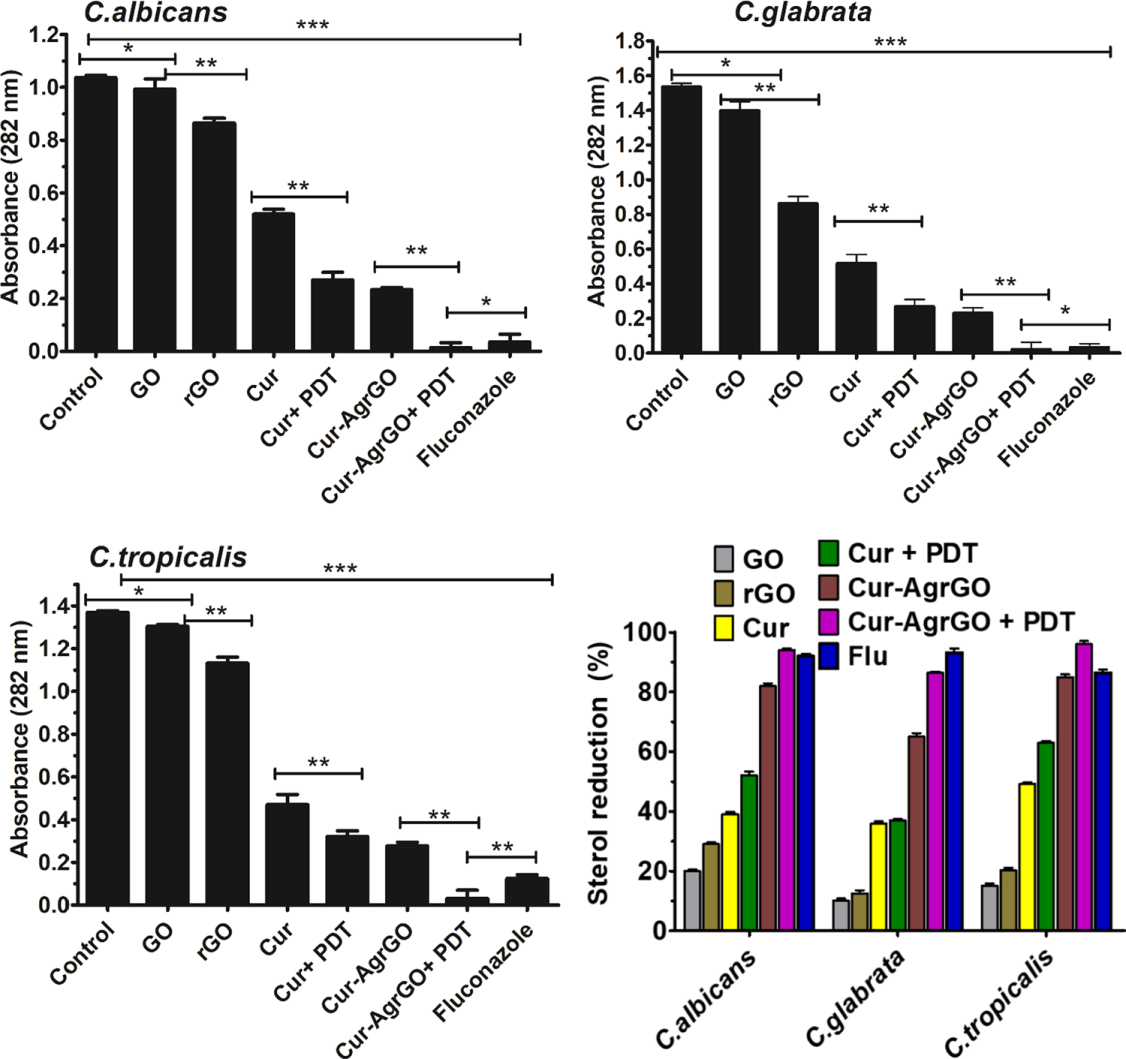
UV spectrophotometric sterol quantification of *Candida* spp. treated with GO, rGO, Cur, Cur + PDT, Cur-AgrGO, Cur-AgrGO + PDT and fluconazole

Figure 9 shows an extensive decrease in total cellular ergosterol content due to drug-based treatments in various *Candida* species. The control groups with untreated cells did not show any changes in ergosterol levels. However, when treated with Cur AgrGO + PDT, significant decreases in ergosterol levels were observed; 96.2% in *C. albicans*, 96% in *C. tropicalis*, and 86.4% in *C. glabrata*. Notably, fluconazole showed effectiveness, resulting in reductions of only 95% in *C. albicans*, 94.4% in *C. tropicalis*, and 84.5%, in *C. glabrata*. Furthermore, exposure to PDT alone and the Cur-AgrGO nanocomposite also led to a significant reduction in ergosterol levels. These results strongly suggest that the nanocomposites primarily target the cell membrane by reducing ergosterol levels, which play a crucial role as a key component of fungal cell membranes. This decline indicates a disruption of membrane integrity and homeostasis, which highlights the potential of nanocomposite-based treatments as strategies for combating fungal infections by disrupting membrane function.

#### Estimation of hydrolytic enzyme secretion

**Fig. 10:**
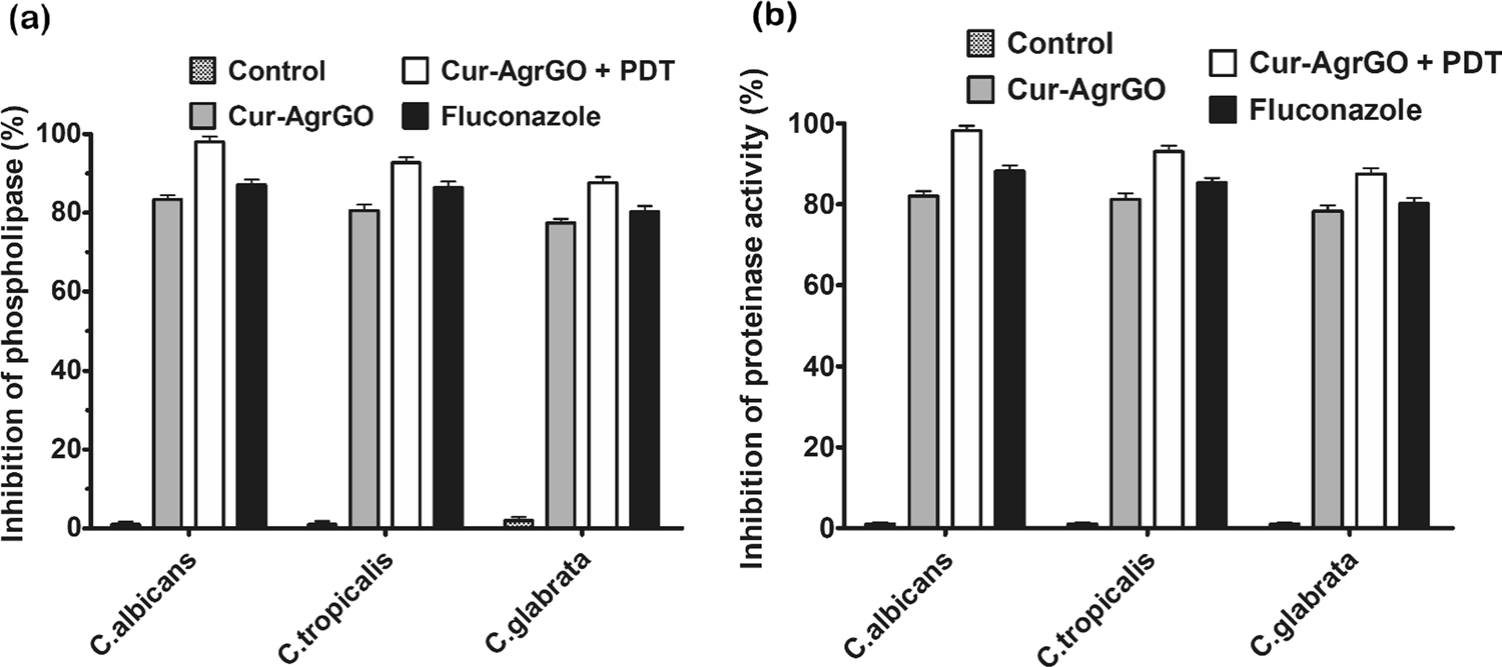
Effect of Cur-AgrGO and Cur-AgrGO + PDT on hydrolytic enzyme secretion. **a** Phospholipase secretion activity of different *Candida* spp. **b** Proteinase secretion activity of different *Candida* spp

*Candida* spp. pathogenicity involves the development of specific virulence factors, such as hydrolytic enzyme release, tissue adhesion, and morphological transformation [54]. The effects of Cur-AgrGO and Cur-AgrGO + PDT at inhibitory levels on hydrolytic enzyme activity were assessed. The enzyme activity was quantified by measuring the zone values (Fig. 10). Cur-AgrGO and Cur-AgrGO + PDT showed a reduction in *C. glabrata*, although their inhibitory impact was relatively low. Enzyme secretion resulted in an increase in zone values for proteinases and phospholipases at MIC, indicating reduced enzyme activity. It is important to note that not all *Candida* strains exhibit high hydrolytic enzyme secretory activity, as fungal pathogenicity is influenced by factors such as morphological changes and biofilm formation [55]. *C. glabrata* demonstrated lower inhibition of proteinase and phospholipase secretion than the other species when exposed to Cur-AgrGO and Cur-AgrGO + PDT. These findings emphasize the complex interplay between hydrolytic enzyme activity and fungal pathogenicity, shedding light on the potential of Cur-AgrGO nanocomposites in modulating virulence factors associated with *Candida* infections.

## Conclusion

In summary, our investigation underscores the considerable potential of carbon-based silver drug nano combinations as potent antifungal agents, particularly in the context of combating multidrug-resistant fungal infections. Through the synthesis and thorough characterization of GO, rGO, and Cur-AgrGO nanocomposites, we have unveiled their robust antifungal efficacy against clinically significant *Candida spp*., including *C. albicans*, *C. glabrata*, and *C. tropicalis*. Our experimental findings, supported by rigorous testing, reveal the promising antifungal effects of these nanocomposites. Moreover, the strategic integration of PDT further amplifies their effectiveness, with Cur-AgrGO combined with PDT demonstrating superior inhibition of virulence traits compared to Cur-AgrGO alone, particularly at the MIC. Mechanistically, our investigations elucidate that the antifungal action primarily involves ROS generation and membrane polarization, with Cur-AgrGO combined with PDT exhibiting heightened effects in these pathways. Notably, this nanocomposite treatment also induces surface damage, compromises cell membrane integrity, and suppresses critical virulence factors such as rapid colonization, hyphal transition, biofilm formation, and hydrolytic enzyme secretion. Taken together, these findings underscore the potential of Cur-AgrGO nanocomposites as promising candidates for the development of novel antifungal drugs in various biomedical applications, offering a potent means to impede the progression of pathogenesis in *Candida spp.* Further research efforts are warranted to refine and optimize these nanocomposites for clinical translation, ultimately contributing to the advancement of therapeutic strategies against multidrug-resistant fungal infections and improving patient outcomes.

## Data Availability

All data supporting the findings of this research are available within the paper.

## References

[1] Nikou SA, Kichik N, Brown R, Ponde NO, Ho J, Naglik JR, Richardson JP (2019). Candida albicans interactions with mucosal surfaces during health and disease. Pathogens.

[2] Zambrano P, Xavier LC, Santos AM, Rossato L, da Costa JC, Serafini MR, Aragón M, Souto RB, Alves IA (2022). What do we have that is new in antifungal peptides? A patent review. Future Microbiol.

[3] Fidel PL, Vazquez JA, Sobel JD (1999). Candida glabrata: review of epidemiology, pathogenesis, and clinical disease with comparison to C-albicans. Clin Microbiol Rev.

[4] Chandra J, Kuhn DM, Mukherjee PK, Hoyer LL, McCormick T, Ghannoum MA (2001). Biofilm formation by the fungal pathogen Candida albicans: development, architecture, and drug resistance. J Bacteriol.

[5] Bohner F, Papp C, Gacser A (2022). The effect of antifungal resistance development on the virulence of Candida species. FEMS Yeast Res.

[6] Hinrichs C, Wiese-Posselt M, Graf B, Geffers C, Weikert B, Enghard P, Aldejohann A, Schrauder A, Knaust A, Eckardt KU, Gastmeier P, Kurzai O (2022). Successful control of Candida auris transmission in a German COVID-19 intensive care unit. Mycoses.

[7] Spettel K, Bumberger D, Camp I, Kriz R, Willinger B (2022). Efficacy of octenidine against emerging echinocandin-, azole- and multidrug-resistant Candida albicans and Candida glabrata. J Glob Antimicrob Resist.

[8] Gutierrez JA, Caballero S, Diaz LA, Guerrero MA, Ruiz J, Ortiz CC (2018). High antifungal activity against candida species of monometallic and bimetallic nanoparticles synthesized in nanoreactors. ACS Biomater Sci Eng.

[9] Bekmukhametova A, Ruprai H, Hook JM, Mawad D, Houang J, Lauto A (2020). Photodynamic therapy with nanoparticles to combat microbial infection and resistance. Nanoscale.

[10] Delavy M, Sertour N, d'Enfert C, Bougnoux ME (2023). Metagenomics and metabolomics approaches in the study of colonization of host niches: a framework for finding microbiome-based antifungal strategies. Trends Microbiol.

[11] Saratale RG, Karuppusamy I, Saratale GD, Pugazhendhi A, Kumar G, Park Y, Ghodake GS, Bharagava RN, Banu JR, Shin HS (2018). A comprehensive review on green nanomaterials using biological systems: recent perception and their future applications. Colloid Surface B..

[12] Balakrishnan D, Lee CI (2022). Surface functionalization of bamboo with silver-reduced graphene oxide nanosheets to improve hydrophobicity and mold resistance. Coatings.

[13] Lei Y, Zhang T, Lin YC, Granzier-Nakajima T, Bepete G, Kowalczyk DA, Lin Z, Zhou D, Schranghamer TF, Dodda A, Sebastian A, Chen Y, Liu Y, Pourtois G, Kempa TJ, Schuler B, Edmonds MT, Quek SY, Wurstbauer U, Wu SM, Glavin NR, Das S, Dash SP, Redwing JM, Robinson JA, Terrones M (2022). Graphene and beyond: recent advances in two-dimensional materials synthesis, properties, and devices. ACS Nanosci Au..

[14] Hiremath N, Kumar R, Hwang KC, Banerjee I, Thangudu S, Vankayala R (2022). Near-infrared light activatable two-dimensional nanomaterials for theranostic applications: a comprehensive review. Acs Appl Nano Mater.

[15] Halbandge SD, Jadhav AK, Jangid PM, Shelar AV, Patil RH, Karuppayil SM (2019). Molecular targets of biofabricated silver nanoparticles in Candida albicans. J Antibiot.

[16] Serrano-Díaz P, Williams DW, Vega-Arreguin J, Manisekaran R, Twigg J, Morse D, García-Contreras R, Arenas-Arrocena MC, Acosta-Torres LS (2023). leaf-mediated synthesis of silver nanoparticles and their transcriptomic effects on Candida albicans. Green Process Synth.

[17] Rodriguez-Cerdeira C, Martinez-Herrera E, Fabbrocini G, Sanchez-Blanco B, Lopez-Barcenas A, EL-Samahy M, Juarez-Duran ER, Gonzalez-Cespon JL (2021). New applications of photodynamic therapy in the management of candidiasis. J Fungi.

[18] Qi ML, Chi MH, Sun XL, Xie XJ, Weir MD, Oates TW, Zhou YM, Wang L, Bai YX, Xu HHK (2019). Novel nanomaterial-based antibacterial photodynamic therapies to combat oral bacterial biofilms and infectious diseases. Int J Nanomed.

[19] Dovigo LN, Pavarina AC, Mima EGD, Giampaolo ET, Vergani CE, Bagnato VS (2011). Fungicidal effect of photodynamic therapy against fluconazole-resistant Candida albicans and Candida glabrata. Mycoses.

[20] Sulaiman C, George BP, Balachandran I, Abrahamse H (2022). Photoactive herbal compounds: a green approach to photodynamic therapy. Molecules.

[21] Sabaghi M, Tavasoli S, Hoseyni SZ, Mozafari MR, Degraeve P, Katouzian I (2022). A critical review on approaches to regulate the release rate of bioactive compounds from biopolymeric matrices. Food Chem.

[22] Rizvi SAA, Kashanian S, Alavi M (2023). Demothoxycurcumin as a curcumin analogue with anticancer, antimicrobial, anti-inflammatory, and neuroprotective activities: micro and nanosystems. Nano Micro Biosyst.

[23] Ehsanifard Z, Motaqi M, Hosseini S, Hasani F (2023). Effects of curcuminoids and resveratrol in micro and nanoformulations on brain-derived neurotrophic factor: a brief review. Micro Nano Bio Aspects.

[24] Hsieh YH, Zhang JH, Chuang WC, Yu KH, Huang XB, Lee YC, Lee CI (2018). An in vitro study on the effect of combined treatment with photodynamic and chemical therapies on candida albicans. Int J Mol Sci.

[25] Hajifathali S, Lesan S, Lotfali E, Salimi-Sabour E, Khatibi M (2023). Investigation of the antifungal effects of curcumin against nystatin-resistant Candida albicans. Dent Res J (Isfahan)..

[26] Sun YM, Zhang HY, Chen DZ, Liu CB (2002). Theoretical elucidation on the antioxidant mechanism of curcumin: a DFT study. Org Lett.

[27] Jiang Y, Leung AW, Hua HY, Rao XC, Xu CS (2014). Photodynamic action of LED-activated curcumin against involving intracellular ROS increase and membrane damage. Int J Photoenergy.

[28] Kumar A, Dhamgaye S, Maurya IK, Singh A, Sharma M, Prasad R (2014). Curcumin targets cell wall integrity via calcineurin-mediated signaling in. Antimicrob Agents Chemother.

[29] Cheraghipour K, Ezatpour B, Masoori L, Marzban A, Sepahvand A, Rouzbahani AK, Moridnia A, Khanizadeh S, Mahmoudvand H (2021). Anti-candida activity of curcumin: a systematic review. Curr Drug Discov Technol.

[30] Cacaci M, Squitieri D, Palmieri V, Torelli R, Perini G, Campolo M, Di Vito M, Papi M, Posteraro B, Sanguinetti M, Bugli F (2023). Curcumin-functionalized graphene oxide strongly prevents candida parapsilosis adhesion and biofilm formation. Pharmaceuticals.

[31] Sharma M, Manoharlal R, Puri N, Prasad R (2010). Antifungal curcumin induces reactive oxygen species and triggers an early apoptosis but prevents hyphae development by targeting the global repressor TUP1 in Candida albicans. Biosci Rep.

[32] Rabiee N, Dokmeci MR, Zarrabi A, Makvandi P, Saeb MR, Karimi-Maleh H, Jafarzadeh S, Karaman C, Yamauchi Y, Warkiani ME, Bencherif SA, Mehta G, Eguchi M, Kaushik A, Shahbazi M-A, Paiva-Santos AC, Ryl J, Lima EC, Hamblin MR, Varma RS, Huh Y, Vilian ATE, Gupta PK, Lakhera SK, Kesari KK, Liu Y-T, Tahriri M, Rama Raju GS, Adeli M, Mohammadi A, Wang J, Ansari MZ, Aminabhavi T, Savoji H, Sethi G, Bączek T, Kot-Wasik A, Penoff ME, Nafchi AM, Kucinska-Lipka J, Zargar M, Asadnia M, Aref AR, Safarkhani M, Ashrafizadeh M, Umapathi R, Ghasemi A, Radisic M (2023). Green Biomaterials: fundamental principles. Green Biomater.

[33] Hsieh YH, Chuang WC, Yu KH, Jheng CP, Lee CI. Sequential Photodynamic Therapy with Phthalocyanine Encapsulated Chitosan-Tripolyphosphate Nanoparticles and Flucytosine Treatment against Candida tropicalis. Pharmaceutics. 2019;11. 10.3390/pharmaceutics11010016 PMC6359070 30621174

[34] Silva-Dias A, Miranda IM, Branco J, Monteiro-Soares M, Pina-Vaz C, Rodrigues AG. Adhesion, biofilm formation, cell surface hydrophobicity, and antifungal planktonic susceptibility: relationship among Candida spp. Front Microbiol. 2015;6. 10.3389/fmicb.2015.00205 PMC4357307 25814989

[35] Soczo G, Kardos G, McNicholas PM, Balogh E, Gergely L, Varga I, Kelentey B, Majoros L (2007). Correlation of posaconazole minimum fungicidal concentration and time kill test against nine Candida species. J Antimicrob Chemother.

[36] Rotilie CA, Fass RJ, Prior RB, Perkins RL (1975). Microdilution technique for antimicrobial susceptibility testing of anaerobic bacteria. Antimicrob Agents Chemother.

[37] Bauer AW, Kirby WM, Sherris JC, Turck M (1966). Antibiotic susceptibility testing by a standardized single disk method. Am J Clin Pathol.

[38] Entradas T, Waldron S, Volk M (2020). The detection sensitivity of commonly used singlet oxygen probes in aqueous environments. J Photochem Photobiol B.

[39] Dovigo LN, Pavarina AC, Ribeiro APD, Brunetti IL, Costa CAD, Jacomassi DP, Bagnato VS, Kurachi C (2011). Investigation of the photodynamic effects of curcumin against. Photochem Photobiol.

[40] Fernandes AR, Prieto M, Sa-Correia I (2000). Modification of plasma membrane lipid order and H+-ATPase activity as part of the response of Saccharomyces cerevisiae to cultivation under mild and high copper stress. Arch Microbiol.

[41] Nett JE, Sanchez H, Cain MT, Ross KM, Andes DR (2011). Interface of Candida albicans biofilm matrix-associated drug resistance and cell wall integrity regulation. Eukaryot Cell.

[42] Arthington-Skaggs BA, Jradi H, Desai T, Morrison CJ (1999). Quantitation of ergosterol content: Novel method for determination of fluconazole susceptibility of Candida albicans. J Clin Microbiol.

[43] El-adly A, Shabana I (2018). Antimicrobial activity of green silver nanoparticles against fluconazole-resistant candida albicans in animal model. Egypt J Bot.

[44] Carradori S, Chimenti P, Fazzari M, Granese A, Angiolella L (2016). Antimicrobial activity, synergism and inhibition of germ tube formation by Crocus sativus-derived compounds against Candida spp. J Enzym Inhib Med Chem.

[45] Li C, Wang X, Chen F, Zhang C, Zhi X, Wang K, Cui D (2013). The antifungal activity of graphene oxide-silver nanocomposites. Biomaterials.

[46] Yang SH, Lee T, Seo E, Ko EH, Choi IS, Kim BS (2012). Interfacing living yeast cells with graphene oxide nanosheaths. Macromol Biosci.

[47] Vazquez-Munoz R, Avalos-Borja M, Castro-Longoria E (2014). Ultrastructural analysis of candida albicans when exposed to silver nanoparticles. PLoS ONE.

[48] Sardi JCO, Scorzoni L, Bernardi T, Fusco-Almeida AM, Giannini MJSM (2013). Candida species: current epidemiology, pathogenicity, biofilm formation, natural antifungal products and new therapeutic options. J Med Microbiol.

[49] Gupta P, Goel A, Singh KR, Meher MK, Gulati K, Poluri KM (2021). Dissecting the anti-biofilm potency of kappa-carrageenan capped silver nanoparticles against Candida species. Int J Biol Macromol.

[50] Andrade MC, Ribeiro APD, Dovigo LN, Brunetti IL, Giampaolo ET, Bagnato VS, Pavarina AC (2013). Effect of different pre-irradiation times on curcumin-mediated photodynamic therapy against planktonic cultures and biofilms of Candida spp. Arch Oral Biol.

[51] Lee DG, Park Y, Kim PI, Jeong HG, Woo ER, Hahm KS (2002). Influence on the plasma membrane of Candida albicans by HP (2–9)-magainin 2 (1–12) hybrid peptide. Biochem Bioph Res Commun.

[52] Khan A, Ahmad A, Khan LA, Manzoor N (2014). *Ocimum sanctum* (L.) essential oil and its lead molecules induce apoptosis in Candida albicans. Res Microbiol.

[53] Nes WR, Sekula BC, Nes WD, Adler JH (1978). The functional importance of structural features of ergosterol in yeast. J Biol Chem.

[54] Taniguchi L, de Fatima FB, Rosa RT, de Paula ECA, Gursky LC, Elifio-Esposito SL, Parahitiyawa N, Samaranayake LP, Rosa EA (2009). Proposal of a low-cost protocol for colorimetric semi-quantification of secretory phospholipase by Candida albicans grown in planktonic and biofilm phases. J Microbiol Methods.

[55] Mattei AS, Alves SH, Severo CB, Guazzelli Lda S, Oliveira Fde M, Severo LC (2013). Determination of germ tube, phospholipase, and proteinase production by bloodstream isolates of Candida albicans. Rev Soc Bras Med Trop.

